# The Development and Validation of the Food Memory Bias Test for a Large-Scale Epidemiological Study in a Multiethnic Asian Population

**DOI:** 10.3390/nu18172912

**Published:** 2026-09-04

**Authors:** Brendon Yi Neng Wong, Irving Yu Le Shua, Rahmania Putri Dewinta, Vedhavaishnavi Sugumaran, Kaleeswaran Shaminidevi, Nadia Loh Ting Wei, Jimmy Lee, Max Lam, John Chambers, Theresia Mina

**Affiliations:** 1 Lee Kong Chian School of Medicine, Nanyang Technological University, Singapore 308232, Singapore; e1523043@u.nus.edu (B.Y.N.W.); shua0002@e.ntu.edu.sg (I.Y.L.S.); rahmaniadewinta25@gmail.com (R.P.D.); vedhavaishnavi05@gmail.com (V.S.); ksnshaminidv@gmail.com (K.S.); jimmy.lee@nhghealth.com.sg (J.L.); max.lam@ntu.edu.sg (M.L.); john.chambers@ntu.edu.sg (J.C.); 2 Department of Economics, National University of Singapore, Singapore 117570, Singapore; 3 Republic Polytechnic, Singapore 738964, Singapore; 4 North Region, Institute of Mental Health, Singapore 539747, Singapore; 5 Precision Health Research (PRECISE), Singapore 139234, Singapore; 6 Department of Epidemiology and Biostatistics, School of Public Health Faculty of Medicine, Imperial College London, London W2 1PG, UK

**Keywords:** eating behaviour, obesity, cognition, memory, bias, nutrition, epidemiology

## Abstract

Background: Memory influences decision-making regarding eating behaviour. However, marketing utilises our memory to exploit our consumptive habits. Whether we have an inherent bias towards memorising visual cues associated with high-calorie foods and how such bias contributes to our health outcomes remains to be determined. We therefore aimed to develop and validate a computerized, image-based Food Memory Bias Task (FMBT) for use in multiethnic Asian populations. Methods: In the development phase involving 172 participants, 91.9% rated the instrument as user-friendly and we optimised the instrument format into 12 visual food cues and 30-min delay period to avoid a ceiling effect. In the validation phase involving 184 multi-ethnic Asian participants (49.3 (14.5) years old, 44.6% male, 71.7% Chinese), FMBT memory score was associated with the brief assessment of cognition memory (β(*p*) = 0.23 (5.1 × 10^−6^)), and general cognition score ‘*g*’ (β(*p*) = 0.30 (6.5 × 10^−9^)). Results: Higher memory bias was associated with lower memory score in the low-calorie bias group (r=−0.33, p=0.002), but not in the hig h-calorie bias group. In this present study, despite randomly administering two versions of the test across visits, the FMBT bias score demonstrates a significant learning effect and low reproducibility (*r*(*p*) = −0.14 (0.11) across visits). Food memory bias score is associated with higher food approach traits according to the Adult-Eating Behaviour Questionnaire (β(*p*) = 0.23(0.015)), independent of age, sex, and ethnicity. Conclusion: FMBT provides an opportunity to address epidemiological questions on how food memory bias influences dietary habit and cardiometabolic health, and how it is in turn shaped by our built environment.

## 1. Introduction

Obesity is one key preventable risk factor [1] for Type 2 diabetes and cardiovascular diseases, amidst the rising global obesity burden in 94% countries over the past 3 decades [2]. Both sedentary lifestyle and dietary intake are important modifiable contributors to obesity. In addition to prescribing meal replacements, caloric restriction protocol, or exercise modules, achieving more sustainable weight loss through behavioural modification has gained prominence, with a recent systematic review of randomised controlled trials reporting comparable efficacy of behavioural interventions compared with pharmacological agents [3]. Defined as internally coordinated responses to food-related internal and external stimuli [4], eating behaviour is constructed through developmental exposure, cognitive processing, and psychological state [5].

The role of memory, particularly episodic memory, in influencing decision-making is long established [6]. Product and service marketing through advertisements encourage future purchases by exploiting our episodic memory [7]. Genetically predicted general cognitive function, including episodic memory, is causally linked with increased BMI [8] and vice versa [9]. Episodic memory influences decision-making processes that involve dietary choices and appetite [10], contributing to individual eating behaviour, and it is a key element in food marketing [11]. Recent behavioural experiments across diverse populations have suggested the presence of human spatial memory bias towards high-calorie food as an evolutionary adaptation strategy for survival [12]. In an experiment involving 41 women, enhanced spatial memory towards high-caloric food items was associated with higher BMI [13]. Food information in working memory was also shown to increase attentional bias towards food, which is enhanced in individuals with obesity [10]. This is of concern, as a biased working or episodic memory towards high-calorie food might promote the vicious cycle of obesity [14].

Recent studies have focused on spatial memory bias towards food items, and none have evaluated the importance of recall memory in influencing dietary intake. Restrictions on high-calorie food advertisements in the UK are estimated to have reduced the prevalence of obesity [15], but whether we possess an inherent bias towards memorising high-calorie food visual cues regardless of health status, and how such bias contributes to dietary intake and health outcomes, remains to be determined. To address these questions, we need to objectively measure food memory bias at population levels using a standard tool. Here, we aimed to develop and perform initial validation on a novel, computerized, image-based Food Memory Bias Task (FMBT) for application in a large-scale population study, with aspiration to monitor population-level variations across ranges of adiposity and secular trends over time. The primary aims of the development phase were to optimise the food image compositions and the delayed recall period in healthy young participants and to evaluate user experience. The primary aims of the validation phase were to compare FMBT with other validated memory tests, describe memory bias, and assess reproducibility. The secondary aims included evaluating the relationship of memory bias with eating behaviour, body composition, and dietary intake; demonstrating memory bias by manipulating the levels of exposure to visual food cues; and performing cultural validation.

## 2. Methodology

### 2.1. The Overview of the Study Design and Participant Recruitment Strategies

**Figure 1:**
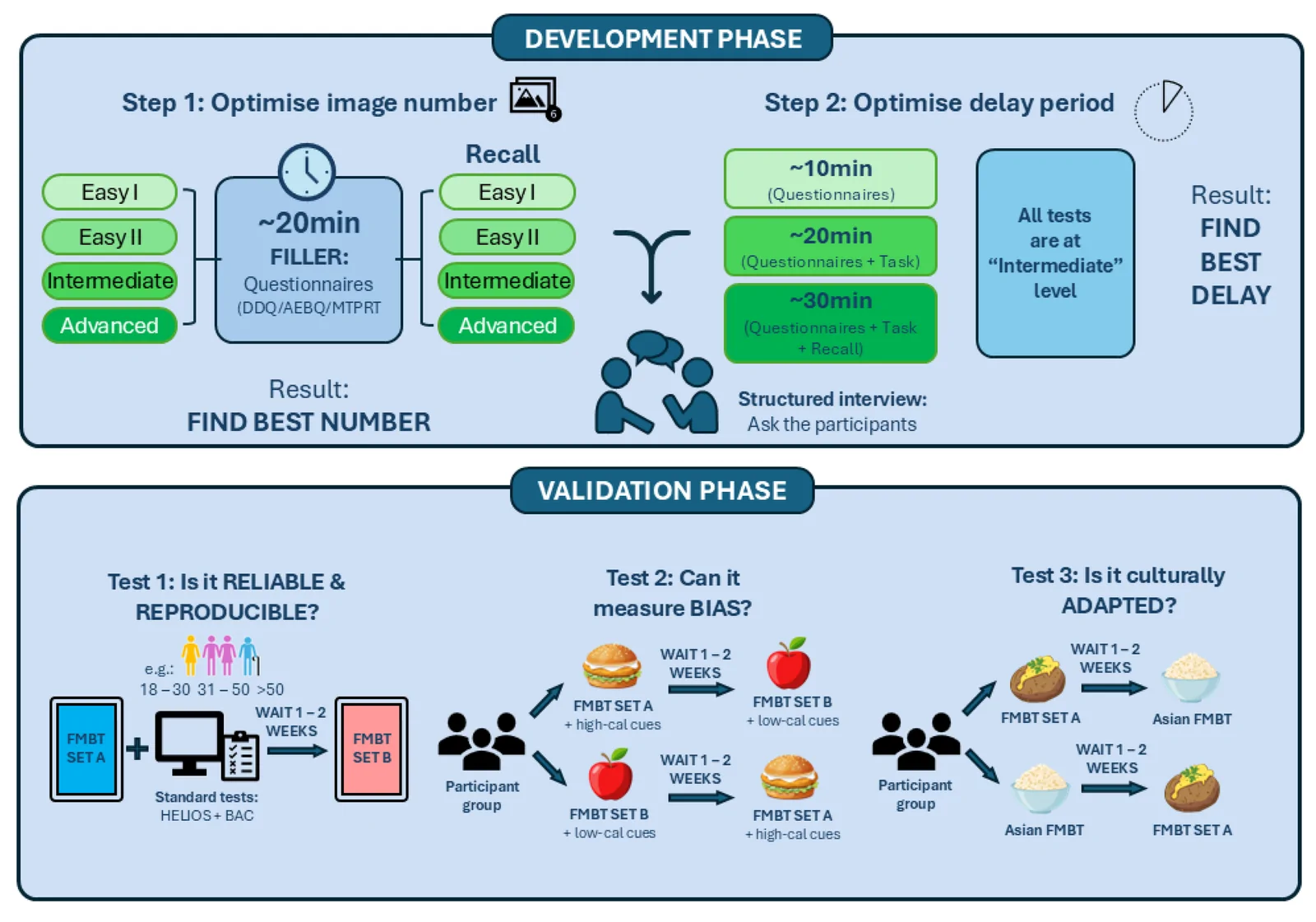
The overall study design of the development and validation of the Food and Memory Bias Test (FMBT). The development phase includes 2 experiments and structured interviews. The advanced (dark green) 30 min combination was adopted into the validation phase. The validation phase comprises broadly 3 experiment modules to achieve 6 aims: (i) compare FMBT with other validated memory tests; (ii) correlate memory performance and age; (iii) assess the associations of bias score with body compositions, other eating behaviour and dietary intake; (iv) demonstrate FMBT ability to assess memory bias through additional visual food cues; (v) ascertain cultural validation; and (vi) demonstrate reproducibility. DDQ = Delayed Discounting Questionnaire; AEBQ = Adult Eating Behaviour Questionnaire; MTPRT = Asian Macronutrient and Taste Preference Ranking Task. Image was created with the help of GeminiAI.

This study was approved by the Nanyang Technological University (NTU) Institutional Regulation Board, Singapore (NTU IRB-2023-671). Figure 1 illustrates the overall study design, encompassing multiple experiments across the development and validation phases. In the development phase, we aimed to optimise image numbers, delay period, and user-friendliness (Figure 1). The inclusion criteria for the development phase were as follows: (i) aged 18–40 years old; (ii) current student or staff in NTU; and (iii) able to read and write in English. The development phase was targeted to recruit 180 study volunteers, including 90 participants for each experimental arm and 30 participants for each sub-experiment. The main rationale for recruiting predominantly young adults in this phase was to ensure that FMBT did not achieve a ceiling effect in a sample of a relatively healthy population.

In the validation phase, we conducted experiments to demonstrate FMBT’s validity and reproducibility. To avoid any learning effect, participants in the development phase were not allowed to participate in the validation phase. The inclusion criteria for the validation phase were as follows: (i) aged 18 and above; (ii) current or past participants of the Health for Life in Singapore (HELIOS) study if ≥30 years old. The HELIOS study is a prospective cohort of a healthy Singapore population comprising individuals aged 30 to 84 years, aiming to understand the aetiology of chronic diseases in multi-ethnic Asian populations [16] (NTU IRB-2016-11-030). We aimed to enrol a total of 250 adults aged 18 and above as part of the FMBT validation phase, anticipating 20% participant dropout. We aimed for proportionate gender (male–female = 1:1) and ethnicity representation (Chinese–Malay–Indian = 3:1:1) (Figure 1). In the bias validation exercise, we enrolled only Chinese participants from the HELIOS study to reduce demographic variations. In the cultural exercise, we recruited only older individuals (>50 years old), as this subgroup was less likely to be familiar with international foods [16].

### 2.2. Developing the FMBT Instrument: Experiment Setup

FMBT was modelled after the delayed recall assessment in the Montreal Cognitive Assessment (MoCA), a globally validated screening test for dementia and mild cognitive impairment [17]. MoCA requires patients to read a list of 5 words in the beginning of the test and deliberately does not inform patients that memorising the words is necessary. After completing other distracting questions, patients need to recall the list of 5 words. There are thus 4 main sections in FMBT: (i) Presentation, (ii) Encoding, (iii) Delay and (iv) Recall (Figure S1A). In the *Presentation* section, we first present a slideshow video of food images, with replay function disabled. We include equal number of high-calorie and low-calorie food images. Next, we ask participants to recall food images in the slideshow (*Encoding*). In the *Encoding* section, the decoy images comprise solely non-food images to ensure easy recall, whereas in the *Recall* section, the decoy comprise both food images and non-food images (Tables S1 and S2). Participants then complete non-mnemonic eating behavioural questionnaires (*Delay*) and recall the images in the slideshow again (*Recall*). Participants can select as few or as many images as required in the *Encoding* and *Recall* sections. Like MoCA, we do not inform participants that memorising the slideshow contents is required, providing an element of surprise to assess implicit memory in the *Recall* section.

To ensure that FMBT does not produce a significant ceiling effect, we optimised the number of images and delay period through 2 experiments. In Experiment #1, we tested 4 difficulty levels with increasing numbers of food images to recall: Easy I, Easy II, Intermediate, and Advanced, while keeping the *Delay* period constant (Figure 1). The total number of images that needed to be memorised were 6, 8, 10, and 12, respectively (Tables S1 and S2). In Experiment #2, we evaluated 3 levels of delay period: 10 min, 20 min, and 30 min, while keeping the number of food images constant (Figure 1). We also performed structured interviews to assess whether participants (i) understood the rationale of the project, (ii) navigated FMBT easily, (iii) were clear with regard to the instructions, and (iv) faced any additional problems.

The *Delay* section comprises eating behavioural questions, including the following: (i) Adult Eating Behaviour Questionnaire (AEBQ) [18], (ii) Delayed Discounting Questionnaire [19], (iii) Asian Macronutrient and Taste Preference Ranking Task [20,21], and (iv) 24 h diet recall [22]. The AEBQ covers 2 opposite psychological responses to food cues: food approach and food avoidance [18]. The AEBQ comprises 35 items answered on a 1 to 5-point Likert scale, with established scoring protocol [18]. The Delayed Discounting Questionnaire is a series of hypothetical choice questions on delaying immediate monetary reward for higher financial gain [19]. The Asian Macronutrient and Taste Preference Ranking Task is a computerized test measuring preferences for macronutrient categories: high-carbohydrate, high-fat, high-protein, low-energy, sweet taste and savoury taste [20,21]. In this context, 24 h diet recall is a very established structured interview technique commonly used in nutrition and dietetics to record food that an individual has consumed in the past 24 h [23]. Altogether, this amounts to ~30 min delay.

### 2.3. Developing FMBT Instruments: Shortlisting Food Images for FMBT

We shortlisted a total of 47 unique food and non-food images from the Food-Pics Extended Database [24], a database of gold-standard food images for psychometric testing purposes, with images presented on a uniform white background, accompanied by nutritional transparency and visual normative scores (Tables S1 and S2). Food items were arranged in ascending order of caloric density to identify natural breakpoints separating low- and high-calorie foods. We define images of foods with ≤53 kCal/100 g as low-calorie (primarily fresh fruits and vegetables) while those with ≥140 kCal/100 g are high-calorie (primarily processed foods). This bimodal approach excludes foods with intermediate calorie levels, thereby increasing the probability of identifying memory bias. We ensured that the macronutrients of the high- and low-calorie foods were significantly different (276 (108) KCal/100 g vs. 23.9 (11.5) KCal/100 g, *p* = 2.62 × 10^−7^, Tables S3 and S4).

The image slideshow was created using Video Editor in Microsoft Photos Legacy, with 2.5 s of display duration per image. The video displayed the food and non-food images in alternate fashion to reduce any bias. We constructed FMBT using Qualtrics (2023–2024, Seattle, WA, USA). We used Windows-based tablets for data (~8.7″, Samsung, Beijing, China). The display orders of images in the *Encoding* and *Recall* sections were randomised.

Singapore has an urban, international food environment and is well known as the Asian gateway for global food and beverages industries; however, as Food-Pics contains relatively few Asian-specific food images, we further identified cultural food images from CROCUFID, a cross-cultural food database for behavioural experiments [25] and additional images from Freepik (https://freepik.com, accessed on 9 September 2023, now known as Magnific^TM^, Málaga, Spain). Figure S2 and Table S5 summarise the shortlisting process to generate a compendium of cultural food images. In total, we identified 183 suitable cultural food images (Figure S2). Table S6 describes the scoring of the Asian FMBT.

### 2.4. Validating FMBT Instruments: Experiment Setup

We conducted 6 experiments across 3 study packages to demonstrate FMBT’s validity and reproducibility: (i) comparing FMBT with other validated memory tests; (ii) evaluating the correlations between memory performance and age; (iii) assessing the associations of FMBT with body compositions, eating behaviour and dietary intake; (iv) demonstrating the modulation of bias scores through additional visual food cues (“bias experiment”); (v) administering the Asian FMBT (“cultural validation”); and (vi) performing reproducibility testing. There were no participant overlaps across the 3 study packages. The main study package included Experiments (i), (ii), (iii) and (vi).

Across all experiments, we administered two locally validated memory screeners, the HELIOS study memory and thinking test [9] and the Brief Assessment of Cognition (BAC) [23] verbal memory section. These tests were administered only on the first visit to avoid learning effect. The order in which participants complete the BAC and HELIOS memory task was randomised. To facilitate Experiments (ii) and (iii), we obtained the age, sex, ethnicity, BMI, and the Food Frequency Questionnaire (FFQ)-based macronutrient intake of current and past HELIOS study participants. We additionally measured the height and weight of non-HELIOS study participants (<30 years old). Dietary intake was available only from the HELIOS study participants.

**Figure 2:**
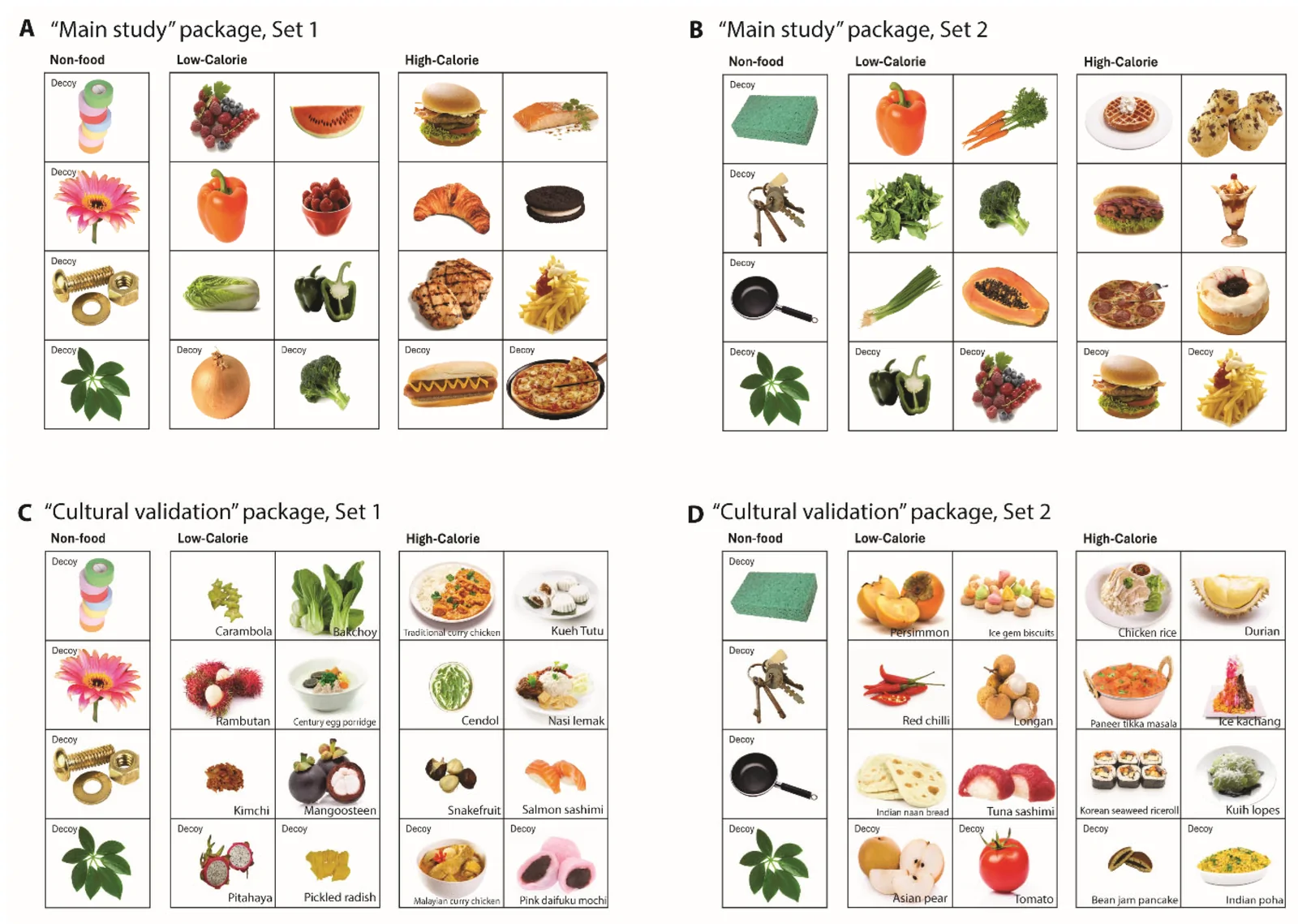
The compilation of food and non-food images used in the FMBT validation phase across study packages. (**A**–**D**) Food images are categorised into low-calorie and high-calorie food items. “Decoy” refers to food images that are not displayed in the slideshow video prior to the *Encoding* section. Each calorie category includes 2 decoys. The display orders of image in the *Encoding* and *Recall* section are completely randomised. (**C**,**D**) The Asian FMBT, or “cultural validation package comprises foods of East Asian (Chinese, Japanese, Korean), Southeast Asian, and South Asian origins. The food names are displayed here to inform the readers who are less familiar with these foods or tropical fruits. Food images displayed during in the *Encoding* and *Recall* section are unlabelled.

**Assessing reproducibility**: All participants returned for a second visit within 2 weeks to assess reproducibility or complete bias experiments. We encouraged participants to synchronise timing across visits. To prevent any learning effect, participants complete FMBT with an alternative picture set on the 2nd visit (Table S4, Figure 2B). The orders of the main or alternative FMBT versions, as well as the main or alternative Asian FMBT versions, were random. Participants were also blinded to the test types. Altogether, the study duration was 20–40 min.

**Modulating food bias**: In Experiment (iv), i.e., the “bias experiment”, we introduced 2 additional visual food cues to mimic the distracting nature of food advertisements to modulate memory bias. The first cue was a non-interactive short video clip (40 s), which was a standardised, social media-style short video featured a hungry person excitedly eating and exclaiming how good the food was (Figure S3). We constructed 2 versions of the standard videos, one containing fruit platter (low-calorie), and another containing burgers and fries (Figure 1 and Figure S3). The second cue was an interactive mock survey to estimate the calorie/serving of food items on a scale of 1 to 100 (Figure S4). The mock survey contained food images in the *Recall* section. The format of the mock survey was adapted from the Food Healthfulness Questionnaire [26,27]. Similarly, we created 2 versions of the mock survey, one containing low-calorie items in the FMBT slideshow, and another containing high-calorie items (Figure 1 and Figure S4). Both visual cues were presented before *Recall* section (Figure S1B). The sequence of high-/low-calorie cues was randomised across visits. Participants were blinded to the types of visual cues.

**Cultural validation**: We administered Asian FMBT for Experiment (v) (Figure 2C,D, Table S6). The Asian FMBT also contains the 4 sections of the FMBT: (i) Presentation, (ii) Encoding, (iii) Delay and (iv) Recall, but with Asian food images instead. There is no video clip or mock survey in Asian FMBT. Across the 2 visits, participants in the “cultural validation” arm completed the “main” FMBT in the first visit, and the alternative Asian version in the subsequent visit, or vice versa (Figure 1).

### 2.5. Deriving FMBT Scores, Cognitive and Other Eating Behavioural Measures

We applied Signal Detection Theory [28,29,30,31,32] to calculate the FMBT score. We categorised food images into 4 quadrants of high- vs. low-calorie vs. true vs. false, respectively (Tables S4 and S6). We defined ***hit rate*** = (*Number of hits selected* + 0.5)/(*total number of hits* + 1) and ***false alarm rate*** = (*Number of false alarm selected* + 0.5)/(*total number of false alarms* + 1) for each calorie group. The addition of 0.5 and 1 is aligned with the Hautus correction for extreme proportions [32]. We then derived the z-score of both ***hit rate*** and ***false alarm rate***. Therefore, ***d’*** = z(***hit rate***) − z(***false alarm rate***) [28,29,30,31] per calorie group, which denotes the participant’s ability to differentiate old and new food images. Subsequently, ***memory*** ***bias score (MBS)*** is ***d’_high-calorie_*** − ***d’_low-calorie_***, where higher score indicates bias towards high-calorie food items, and ***memory score*** (***MS***) is (**(*d’_high-calorie_ + d’_low-calorie_*)/2**).

For the downstream validation analysis, we dimensionally reduced the HELIOS study cognitive test raw scores into ‘***g***’, a proxy of general cognition, as previously described [9]. We then calculated the score for verbal memory according to the previous local validation [33] and the Food Approach and Food Avoidant scores from AEBQ [24]. Finally, we extracted self-reported macronutrient intake from the FFQ of the HELIOS study participants. Macronutrient intake was expressed as the percentage of total energy intake by applying Food and Agricultural Organization-recommended energy density (proteins, carbohydrates, starch, and sugar = 4 Kcal/g, fats = 9 Kcal/g, and fibre= 2 Kcal/g [34]). DDQ, MTPRT, and 24 h diet recall data were used for other independent validation exercises, and they are thus not included in this manuscript.

### 2.6. Statistical Analysis

Statistical analysis and graphical illustrations were performed using R version 4.40, and Adobe Illustrator CS6. In the validation study, we included datasets of participants who completed both visits and had also completed at least 1 memory screener. Prior to any Pearson correlations, we visually inspected histograms to ascertain the assumptions of normality. All continuous variables were z-normalised before regressions. A *p*-value of ≤0.05 was considered statistically significant.

To validate the mnemonic property of FMBT, we derived unadjusted Pearson’s correlations and associations of ***MS*** with BAC verbal memory and ‘***g***’, with associations adjusted for sex, age, ethnicity, study visits, and study experiments. We also performed multivariable linear regressions of ***MS*** with age before and after adjusting for covariates. Post hoc model diagnostics confirmed acceptable multicollinearity (VIF < 6.5), minimal leverage (Cook’s D < 0.10), and valid OLS parameter estimation. To assess learning effect, we determined whether absolute difference in ***MS*** across visits was correlated with BAC verbal memory, ‘***g***’, and age.

To understand the distribution of ***MBS***, we examined the histogram of ***MBS***. Next, to understand the relationship of ***MBS***, ***MS***, and age while accounting for non-linear distribution, we grouped participants into high-calorie (***MBS*** > 0) and low-calorie (***MBS*** < 0) bias groups, and we examined correlations of absolute ***MBS*** with ***MS*** and age. Subsequently, we derived the Bland–Altman plots and calculated the Pearson correlation of FMBT scores across study visits from the main study to determine reproducibility. We also performed paired *t*-test of ***MBS*** across visits to detect learning effect.

To demonstrate the instrument’s ability to measure food memory bias score, we compared the within-participant ***MBS*** after receiving high-calorie versus low-calorie visual cues, using a paired *t*-test. As part of the cultural validation, we compared within-participant ***MBS*** after completing Asian vs. main FMBT. Finally, we extracted the unadjusted Pearson correlations of ***MBS*** from the “main” study package with eating behaviour, self-reported macronutrient intake variables, and BMI, and associations after adjusting for sex, age, and ethnicity.

## 3. Results

### 3.1. Insights from the Development of FMBT

Figure S5 summarises the screening and enrolment of the development phase; we enrolled a total of 172 participants (~28.7/experiment pool, Figure 1, development phase) and 161 (93.6%) agreed to complete the user interface evaluation. The participants were 52.3% female, 82% Chinese, with an age range ratio (18–30:31–40) of 72:14. Most participants (77.6%) cited that the aim of the test was to investigate memory, followed by food preferences (60.2%) and emotion (11.8%), or combinations of the three. Most participants (91.9%) found the test easy to navigate. A small number of participants (5.0%) missed the start of the slideshow video, despite a warning statement that “*the video would only be played once*”. To alleviate this issue, we incorporated additional slides at the start and the end of the slideshow, with the following texts: “*ATTENTION. The slideshow starts now*” and “*This is the end of the slide*”, respectively.

In this pool of young adults, we did not observe material changes in the ***MS*** across increasing numbers of food images (*p* = 0.999, Figure S6A), nor with increased duration of the *Delay* section (*p* = 0.498, Figure S6B). Increasing the number of images did not increase recall duration (*p* = 0.999, Figure S6C) but a longer delay period did prolong recall duration (*p* = 0.002, Figure S6D). Therefore, to ensure there would be no ceiling effect when moving forward, we adopted the highest number of food images tested (a total 16 images, comprising 12 true images and four decoy images) and the longest duration for the *Delay* section (30 min). This means that the ***MS*** ranges from –2.43 to 2.43, where the maximum value means accurately identifying all the pictures of food shown in the *Presentation* section and not selecting any decoy images during *Recall* section. ***MBS*** thus ranges from −4.87 to 4.87, where the maximum plausible ***MBS*** means selecting all the hits (*n* = 6) for the high-calorie food options and all the decoys for the low-calorie food options and selecting none of the decoys and hits for the high- and low-calorie food images, respectively.

### 3.2. Participant Demographics in the Validation Phase

Overall, we obtained 184 datasets from participants who completed two FMBT study visits and at least one memory screener and had BMI measured (Figure S5). Macronutrient intake was available in a subset of 143 (77.7%) participants (Figure S5). The participants were 49.3 (14.5) years old, 44.6% male participants, 71.7% Chinese, and 89.1% were recruited from the HELIOS study (Table 1). The sex proportion and average BMI were similar across the three validation study packages (Table 1). As intended, participants’ overall age was older in the cultural validation (Table 1). The ***MS***, but not ***MBS***, differed significantly across study packages (Table 1).

### 3.3. Comparison of FMBT Memory Score (MS) with Cognitive Instruments and Age

**Figure 3:**
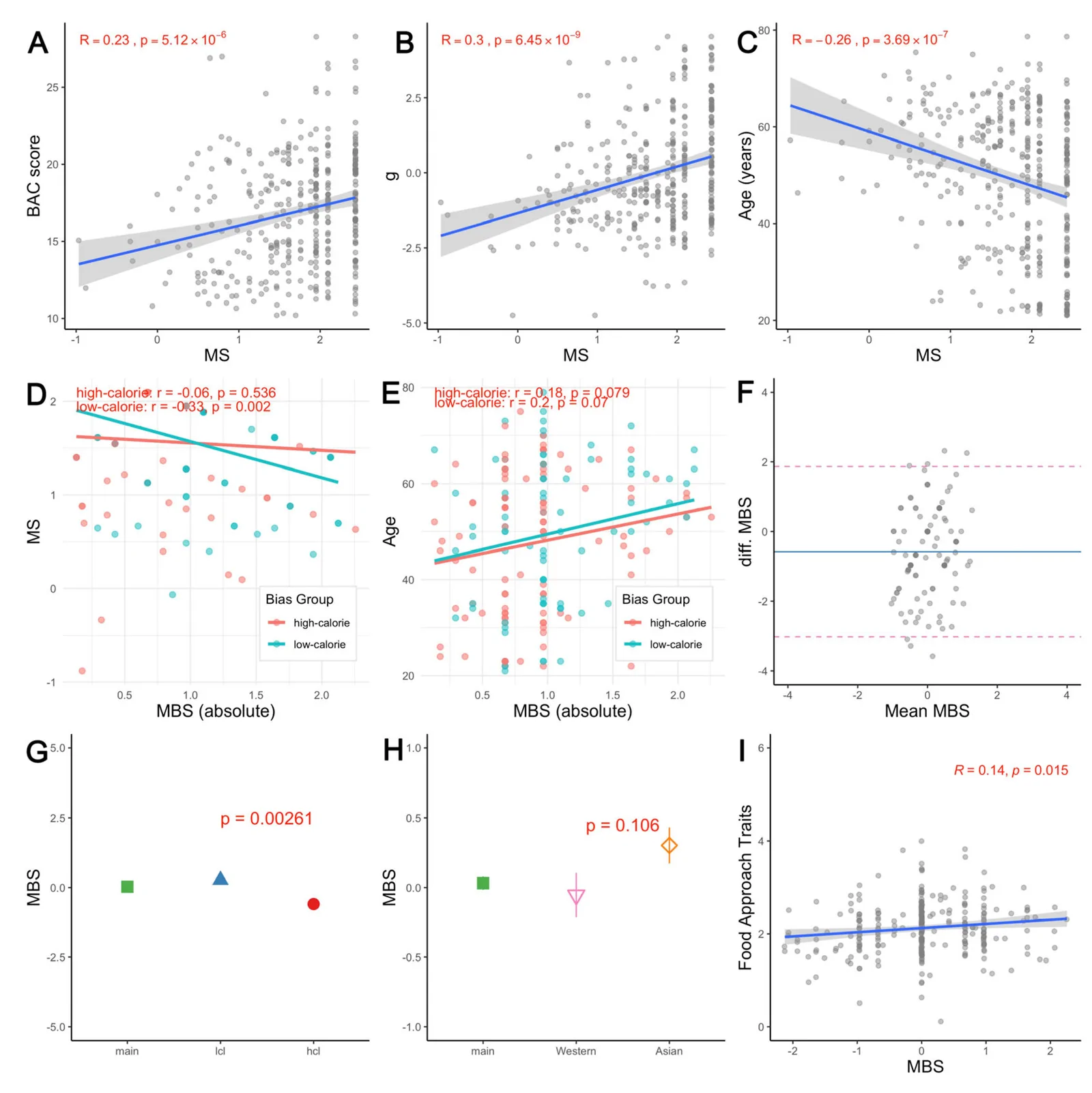
Findings from the multiple experiments to determine the validity of FMBT. Scatterplot of (**A**) BAC verbal memory score, (**B**) general cognitive function ‘***g***’, and (**C**) age with ***MS*** on all available validation study datasets. The linear regressions were adjusted for sex, age, ethnicity, study visit, and study experiments. (**D**) Scatterplot of ***MBS*** and ***MS*** and (**E**) ***MBS*** with age, where high- and low-calorie refers to ***MBS*** > 0 or <0. (**F**) The Bland-Altman plots of ***MBS***. (**G**) The mean (SE) of within-person ***MBS*** across bias experiment; “main study package” has no additional visual cues and is displayed as reference point. (**H**) The mean (SE) of within-person ***MBS*** across “main study package”, the Western vs. Asian FMBT. (**I**) Correlations of ***MBS*** with Food Approach trait from AEBQ.

***MS*** was correlated with BAC verbal memory, ‘***g***’, and age (Pearson’s r(*p*) = 0.23 [95%Cl: 0.14, 0.33] (5.1 × 10^−6^), 0.30 [95%Cl: 0.20, 0.39] (6.5 × 10^−9^), and −0.26 [95%Cl: −0.35, −0.16] (3.7 × 10^−7^), respectively). ***MS*** remained positively associated with BAC verbal memory score (β(*p*) = 0.23 [95%Cl: 0.14, 0.33] (5.1 × 10^−6^)), and ‘***g***’ (β(*p*) = 0.30 [95%Cl: 0.20, 0.39] (6.5 × 10^−9^)), after adjusting for sex, age, ethnicity, study visits, and study experiments (Figure 3A,B). ***MS*** was also inversely associated with age after adjusting for covariates (β(*p*) = −0.26 [95%Cl: −0.35, −0.16] (3.7 × 10^−7^), Figure 3C). Notably, participants with higher BAC verbal memory and ‘***g***’ score, and who were older, had less discrepancy in ***MS*** across visits (Pearson’s r(*p*) with Δ***_MS_*** _across visits_= −0.32 [95%Cl: −0.46, −0.15] (0.0003), −0.26 [95%Cl: −0.42, −0.10] (0.002), and 0.16 [95%Cl: −0.08, 0.32] (0.06), respectively), implying a possible learning effect. The average FMBT completion time was 33.5 (23.25) minutes, and we also found that longer completion time was correlated with lower ***MS*** (r = −0.138, 95% CI: −0.25 to −0.02, *p* = 0.021).

### 3.4. Participants’ Memory Bias Score (MBS) and Potential Learning Effect

The distribution of ***MBS*** appears trimodal, with most participants presenting 0 scores (neutral), and values <0 and >0 denoting low-calorie and high-calorie bias, respectively (Figure S7). In participants with low-calorie food bias (***MBS*** < 0), higher ***MBS*** was correlated with lower ***MS*** (Figure 3D); however, in participants with high-calorie food bias (***MBS*** > 0), ***MBS*** was uncorrelated with ***MS*** (Figure 3D). Additionally, regardless of their bias groups, there was a positive trend between ***MBS*** and age (Figure 3E), suggesting that memory bias was stronger in older individuals.

The Bland–Altman plot of ***MBS*** across 2 weeks shows that most observations were within the limit of difference. The mean bias between visits was −0.576 (95% CI [−0.789, −0.364]). The 95% limits of agreement ranged from −3.017 [−3.385, −2.649] to 1.864 [1.496, 2.232], with a proportional bias *p*-value of 0.0505. However, the Pearson correlation across two visits was not significant −0.14 [95%Cl: −0.31, 0.032] (0.11). Despite randomising two versions of FMBT across visits, the paired *t*-test demonstrated a significant learning effect; ***MBS*** was lower at the second visit by 0.57 points (*p* = 5.37 × 10^−7^), again implying a learning effect. The ${\mathrm{ICC}}_{3,1}$ yielded a low value (${\mathrm{ICC}}_{3,1}\approx 0.00$), reflecting variable individual learning effects across visits. The standard error of measurement (SEM) was 0.88, yielding a minimal detectable change of 2.44.

### 3.5. Food Memory Bias: Insights from Secondary Experiments

Interestingly, in the “bias experiment” with visual food cues to imitate food advertisements, we observed opposite patterns of within-person ***MBS***; bias towards high-calorie foods was higher when low-calorie foods visual cues were shown, and vice versa (*p* = 0.00261, Figure 3G). There were no significant within-person ***MBS*** differences in “main” FMBT version compared with Asian FMBT (*p* = 0.106, Figure 3H). Furthermore, BMI was correlated with higher food-approach traits and self-reported saturated fatty acids and protein consumptions, and lower food-avoidance traits (Table 2). ***MBS*** was correlated with food-approach traits (r(*p*) = 0.14 [95%Cl: 0.028, 0.25] (0.015), Table 2) but not correlated with self-reported dietary intake or BMI (Table 2). After accounting for covariates, food bias remained associated with food-approach traits (β(*p*) = 0.23 [95%Cl: 0.044, 0.41] (0.015), Table 2).

## 4. Discussion

In this study, we developed and validated a Food Memory Bias Task (FMBT) that is suitable for use in a large-scale epidemiological study particularly in multi-ethnic Asian populations. We optimised the numbers of images and delay period to reduce ceiling effect. We then demonstrated the mnemonic properties of FMBT and the relationship of food memory bias with food approach traits, performed cultural validation, and evaluated the reproducibility of the test.

In the development phase, we found that the longest delay period (30 min, 10 images) produced longer recall duration amongst the young adult participants. While increasing the number of food images and the delay period is possible, our goal is to administer FMBT for general population via tablets; therefore, we prioritised minimising participant burden, especially among older adults, and ensured that food images are sufficiently large at 100% magnification. Informing participants that the slideshow will be shown only once may prompt them to instinctively try to memorise the food images; we anticipated very high scores during *Encoding*; however, FMBT avoids informing participants that they need to memorise the slideshow contents until the end of the study, providing an element of surprise when assessing memory in the *Recall* section.

We report consistent positive associations between the FMBT memory scores, the BAC verbal memory score [23,33], and the general cognitive function score ‘***g***’, which incorporates numeric-based and non-food image-based memories [9]. We also observed an inverse association between FMBT memory scores and age. Altogether, these experiments show that FMBT was able to elicit memory responses through food visual cues. Although we applied an alternative set of food images in the second visit, we observed a learning effect particularly amongst younger participants and/or those with higher baseline BAC verbal memory and ‘***g***’. This was possibly contributed to by the diminished element of surprise and a short reproducibility period within 2 weeks. The observed learning effect may limit future test–retest reliability; instead of slideshow formats, future studies should explore alternative methods to introduce image cues, such as through standardised video clips of food commercials or unrelated interactive surveys, as introduced previously in the bias experiments. We also observed variable completion time in the more diverse samples compared with healthy young adults during the development phase. As this could potentially affect not only memory score but also memory bias score, future studies could standardise the *Delay* period by substituting 24 h dietary recall with other more static instruments.

In this validation study recruiting from a population health cohort [16], most participants displayed neutrality in food memory. We found that higher memory bias score was correlated with lower memory score in the low-calorie bias group (***MBS*** < 0), but not in the high-calorie bias group. This discrepancy could suggest that a high-calorie memory bias (***MBS*** > 0) could have a preservation impact on visual food cues. Although cross-sectional and modest, this finding seems aligned with the adaptive foraging theory [12], which postulates that human memory systems are fundamentally biased toward prioritising fitness-relevant, energy-dense survival resources. Our finding supports the observed attentional bias towards high-calorie food when repeated food stimuli are shown [35]. Furthermore, this observation also appears aligned with the neurological literature demonstrating that high-calorie food cues trigger conditioned associations and reinforcement learning through enhanced hippocampal memory consolidation via dopamine pathways [36]. Given this exploratory nature of this sub-group analysis, future studies should carry out further investigations to test this effect in a larger cohort.

Compared with traditional paradigms assessing food-related attentional bias such as the food visual probe task [37,38] or the food Stroop task [39], FMBT provides a distinct, complementary opportunity to examine how food cues are processed and retained as memory. Unlike food go/no-go tasks that assess motor-inhibitory control in the presence of food stimuli [40] or a food spatial memory paradigm that evaluates location recall for calorie-dense items [41], FMBT is designed to capture differential bias toward food high- vs. low-calorie-density food through cognitive encoding. This positions FMBT as a bridge between initial attentional and inhibitory processes with longer-term cognitive representations that drive downstream dietary habits.

Despite inverse correlation between memory performance and age, FMBT also shows that food memory bias, regardless of whether it is high-calorie or low-calorie, is stronger in older individuals. This observation aligns with socioemotional selectivity theory and the supporting fMRI evidence, where older adults exhibit a selective allocation of neural resources, maintaining robust activation within the amygdala and frontoparietal networks during the encoding of salient, motivationally relevant stimuli, even as neural processing of neutral objects sharply declines [37]. Interestingly, despite randomising FMBT instrument versions across visits and framing the study as a study to understand general eating behaviours in a population, our findings point towards social desirability bias. The results of our “bias experiment” exhibited reverse patterns, although this would require independent replication. Second, our reproducibility testing demonstrated a strong learning effect and when subjected to the ‘Asian’ version of FMBT, which was perhaps perceived as neutral, the intra-person score was highly consistent. Following the reduced cognitive load in the second visit and the diminished element of surprise observed earlier, participants plausibly aligned their performance with perceived societal expectations regarding dietary health or memory capability.

We found an association between greater memory bias towards high-calorie food and food-approach traits, though not with dietary intake and BMI. Although we cannot rule out insufficient statistical power due to the small number of individuals with obesity, BMI remains correlated with food-approach and -avoidance traits and protein intake in expected directions. Regardless, few studies have explored the relationship of food memory bias and obesity, although memory and BMI are increasingly understood to be linked through the gut–brain axis [38]. To address the hypothesis that food memory bias is associated with adverse dietary intake and higher BMI, future studies would require larger population samples, ideally with larger numbers of individuals with obesity.

Our study has several limitations. Our instrument is currently only available in English, although language availability can be easily addressed in future through forward/backward translation by professional translators and structured interviews to ascertain clarity. Due to logistical constraints, we were also unable to extend the study period beyond 2 weeks to overcome the learning effect and social desirability bias. We also did not restrict our recruitment to individuals with no obesity or other prevalent cardiometabolic diseases; hence, we could not rule out individuals who had received health interventions that affected their eating behaviour. In this present study, we also observed low memory bias reproducibility due to the significant learning effect and variable delay period. Although this is an exploratory validation study, we acknowledge that there may be increased risk of Type 1 error as we reported multiple associations across several outcomes. We also did not apply linear mixed-effects models in lieu of paired *t*-tests; future studies could consider mixed-effects modelling in broader analysis.

Nevertheless, this study also has several strengths. We intended for the instrument to be applied to large-scale population studies through digital data collection tool. We also attempted cultural validation, widening potential applications of the tool to over 60% of the global Asian diaspora, although we acknowledge the predominantly Chinese participants in this study and that future external validation outside of Singapore is warranted. Designed for tablet and mobile use, our tool is also well-suited for low-resource settings. This reflects broader digital trends, where smartphones drive over 60% of global internet traffic and maintain high penetration rates in low- and middle-income countries [42].

## 5. Conclusions

In this study, we developed a computerized, image-based Food Memory Bias Task (FMBT) and validated the instrument’s mnemonic and bias properties through additional visual cues, performed cultural validation and reproducibility testing. This instrument may provide an opportunity to address fundamental questions on the biological underpinnings of food memory bias and its impacts on dietary habit and cardiometabolic health. Future studies should address the significant learning effect and improve the test–retest reliability by introducing food images using alternative methods in the subsequent visit, and/or reduce the variability in the delay period.

## Figures and Tables

**Table 1:** The general demographics of the validation phase of the Food Memory Bias Test (FMBT).

General Demographics	Overall, *n* = 184	Main, *n* = 129	Bias, *n* = 29	Culture, *n* = 26	P_ANOVA_
Age (years), mean (SD)	49.5 (14.5)	45.6 (15.1)	54.8 (7.44)	61.9 (5.7)	**2 × 10^−8^**
Male, *n* (%)	82 (44.6)	62 (48.1)	11 (37.9)	9 (34.6)	0.369
Ethnicity, Chinese, *n* (%)	132 (71.7)	83 (64.3)	29 (100%)	20 (76.9)	**0.001**
Recruitment source, HELIOS participants, *n* (%)	164 (89.1)	109 (84.5)	29 (100%)	26 (100%)	**0.0004**
BMI (Kg/m^2^), mean (SD)	24.0 (4.1)	24.0 (4.0)	24.2 (4.7)	23.8 (3.7)	0.933
across 2 visits, recall section					
***MS***, mean (SD)	1.71(0.67)	1.8 (0.6)	1.38 (0.8)	1.62 (0.7)	**4.76 × 10^−5^**
***MBS***, mean (SD)	0.013(0.9)	0.03 (0.9)	−0.16 (1.1)	0.12 (0.8)	0.212

Non-Chinese ethnic groups include Malay, Indian and other ethnic groups as verified against participants’ national identity card. The main recruitment source was HELIOS study participants, followed by university students and staff for individuals aged <30 years old. P_ANOVA_ compares the values across the 3 validation study packages. ***MS*** = memory score; ***MBS***: memory bias score.

**Table 2:** The unadjusted relationship of FMBT food bias score with BMI, eating behavioural domains, and dietary intake scores in the main validation study package.

Body Composition	Mean (SD)	R [95% Cl] (*p*) with BMI	R [95% Cl] (*p*) with *MBS*	β(*p*) [95% Cl] with *MBS*
BMI, Kg/m^2^	24.2 (3.7)	—	0.035 [−0.080, 0.15] (0.55)	0.0078 [−0.018, 0.033] (0.55)
**Adult Eating Behaviour Questionnaire**				
Food-approach traits	2.1 (0.7)	**0.158 [0.043, 0.27] (0.007)**	**0.14 [0.028,0.25] (0.015)**	**0.23 [0.045, 0.41] (0.015)**
Food-avoidance traits	1.9 (0.5)	**−0.186 [−0.30, −0.073] (0.001)**	0.030 [−0.085, 0.15] (0.61)	0.056 [−0.16, 0.27] (0.61)
**FFQ-based macronutrient intake**				
Carbohydrates, % Kcal	44.9 (9.0)	−0.025 [−0.16, 0.11] (0.71)	−0.023 [−0.16, 0.11] (0.73)	−0.0024 [−0.016, 0.012] (0.73)
Saturated fatty acids, % Kcal	13.5 (3.0)	**0.134 [0.0015, 0.26] (0.047)**	−0.031 [−0.16, 0.10] (0.65)	−0.010 [−0.053, 0.033] (0.65)
Protein, % Kcal	16.3 (3.1)	**−0.175 [−0.30, −0.044] (0.0094)**	0.034 [−0.099, 0.17] (0.62)	−0.010 [−0.020, 0.050] (0.62)
Fibre, % Kcal	1.7 (0.4)	−0.127 [−0.26, 0.0058] (0.061)	0.042 [−0.091, 0.17] (0.53)	0.094 [−0.20, 0.39] (0.53)

We considered the scores from the “main” validation study packages only, as the additional visual cues in the bias experiments might have affected the relationships of FMBT scores with other variables. Unadjusted correlations with BMI were included as comparison. FFQ = Food Frequency Questionnaire. Covariates include age, sex, ethnicity, ***MS***, and study experiments. ***MS*** = memory score; ***MBS***: memory bias score.

## Data Availability

The anonymised FMBT data and basic demographics in this study will be made open-access via DR-NTU. Any data that were derived following data linkage with the HELIOS Study are not publicly available; data access requests to the HELIOS study can be submitted to the HELIOS Data Access Committee by emailing helios_science@ntu.edu.sg for details.

## Supplementary Materials

The following supporting information can be downloaded at: https://www.mdpi.com/article/10.3390/nu18172912/s1, Figure S1: The diagram of FMBT questionnaire flow; Figure S2: The shortlisting of cultural food images used in the Asian FMBT. Figure S3: A collage of non-interactive short video clips resembling food advertisements. Figure S4: Screenshots of interactive mock survey to estimate the calorie/ serving of food items. Figure S5: The CONSORT diagram of participants for the FMBT development and validation study. Figure S6: Insights from the Development of FMBT. Figure S7: Distribution of memory bias scores. Table S1: The breakdown and description of food images across experimental set-up to optimise the numbers of food images in the Development phase of FMBT. We created an alternative set for each set-up. Note that the alternative set was not administered during the Development phase. Table S2: The breakdown and description of food images across experimental set-up to optimise the delay period in the Development phase of FMBT. Note that the alternative set was not administered during the Development phase. Table S3: The nutrient profile of food images for various experiments in the FMBT development phase. The nutrient profile was extracted from the FoodPics macronutrient data. Table S4: The description of food images for various experiments in the FMBT validation phase. The nutrient profile was extracted from the FoodPics macronutrient data. Table S5: The shortlisting of cultural food images for the developmand and validation of Asian FMBT. The 183 final images include the 189 listed images after exclude images containing raw food items of ingredients. Table S6: The description of food images for various experiments in the Asian FMBT for cultural validation phase.
